# Supplementary material for: Reverse transcription recombinase polymerase amplification-lateral flow assay for detection of pathogenic orthoflaviviruses in mosquito vectors
Source: PeerJ. 2025 Aug 26;13:e19820. doi: 10.7717/peerj.19820 (PMC12396212; doi:10.7717/peerj.19820)
Supplement: Supplemental Information 1 [file peerj-13-19820-s001.pdf]

Table S1: Primers used in this study.

Primer	Sequence (5' - 3')	Ta (°C)	Product size (bp)	Reference
FlaviPath-F †	[5'Dig]AARGGHAGYMGNGCHATHTGGTWYATGTGG-3'	37-40	121	This study
FlaviPath-R ‡	[5'FITC]CCTTCHACWCCDCYBHVDGARTTYTYHCKV-3'			This study
XF-F2	AARGGMAGYMGNGCHATHTGGT	54	215	Xue et al. (2021)
XF-R	GTRTCCCANCCDGCDGTRTCATCNGC			
T7-DENV2 NS5-F	TAATACGACTCACTATAGGGACAAGTCGAACAACCTGGTCCAT	60	197	Modified from Richardson et al. (2006) Patramool et al. (2013)
DENV2 NS5-R	GCCGCACCATTGGTCTTCTC			
DENV-2 NS5-F	ACAAGTCGAACAACCTGGTCCAT	60	177	Richardson et al. (2006)
DENV-2 NS5-R	GCCGCACCATTGGTCTTCTC			
ZIKF	AARTACACATACCARAACAAAGTGGT	60	102	Han et al. (2018)
ZIKR	TCCRCTCCCYCTYTGGTCTTG			

IUB codes for degenerate bases: M=A/C; R=A/G; W=A/T; S=G/C; Y=C/T; K=G/T; V=A/G/C; H=A/C/T; D=A/G/T; B=G/C/T; N=A/G/C/T

†Binding region 8975–9004 bp in NS5 region (8774–11609 bp) of Dengue virus type 1 clone 45AZ5, complete genome (U88536)

‡Binding region 9067–9096 bp in NS5 region of Dengue virus type 1 clone 45AZ5, complete genome (U88536)

References

Han, Y., Mesplède, T., Xu, H., Quan, Y. and Wainberg, M.A. 2018. The antimalarial drug amodiaquine possesses anti-ZIKA virus activities. Journal of Medical Virology 90(5), 796-802.

Patramool, S., Bernard, E., Hamel, R., Natthanej, L., Chazal, N., Surasombatpattana, P., Ekchariyawat, P., Daoust, S., Thongrungkiat, S., Thomas, F., Briant, L. and Missé, D. 2013. Isolation of infectious chikungunya virus and dengue virus using anionic polymer-coated magnetic beads. Journal of Virological Methods 193(1), 55-61.

Richardson, J., Molina-Cruz, A., Salazar, M.I. and Black, W.t. 2006. Quantitative analysis of dengue-2 virus RNA during the extrinsic incubation period in individual *Aedes aegypti*. Am J Trop Med Hyg 74(1), 132-141.

Xue, Z., Zhao, N., Wang, J., Song, X., Meng, F., Liang, W., Zhou, J., Wang, D., Zhang, Z. and Liu, Q. 2021. Establishment and Application of Heminested RT-PCR Assay for Detection of Mosquito-Borne Flavivirus - Guizhou Province, China, 2018. China CDC weekly 3(1), 4-9.

Table S2: Synthetic target oligonucleotide sequences used in this study.

Name	Sequence (5'-3')	bp	Accession Number
Den2Th	GGCAAAAGGCAGCAGAGCCATATGGTACATGTGGCTCGGAGCTCGCTTCCTGGAGTTTGAAGCCCTAGGATTCCTAAATGAAGATCACTGGTTCTCCAGAGAGAACTCCTTGAGTGGAGTGGAAGGAGAA	130	FJ906958.1
Lammi	AGCCAAAGGTAGCAGGGCGATCTGGTACATGTGGCTGGGGGCGAGATACCTCGAGTTTGAAGCGCTGGGGTTCCTAAACGAAGACCATTGGCTGTCAAGGGACAACTCCAAAGGTGGTGTAGAAGGAATT	130	KC692068.1
JEV	AGCTAAAGGAAGCAGGGCCATTTGGTTCATGTGGCTTGGAGCACGGTATCTAGAGTTTGAAGCTTTGGGGTTCCTGAATGAAGACCATTGGCTGAGCCGAGAGAATTCAGGAGGTGGAGTGGAAGGCTCA	130	M18370.1
ZikT	GGCCAAGGGCAGCCGCGCCATCTGGTATATGTGGCTAGGAGCTAGATTTCTAGAGTTCGAAGCCCTTGGATTCTTGAACGAGGATCACTGGATGGGGAGAGAGAACTCAGGAGGTGGTGTTGAAGGGCTG	130	KY272987.1
WNV	AGCTAAAGGCAGCAGAGCCATCTGGTTCATGTGGCTGGGGGCCCGCTTCCTGGAGTTTGAAGCTCTCGGATTCCTCAATGAAGACCACTGGCTGGGTAGGAAGAACTCAGGAGGAGGAGTTGAAGGCTTA	130	M12294.2
Nakiwogo	GCTAAAGGGTCACGTACCATATGGTACATGTGGCTCGGAAGCCGGTTTTTGGAATACGAGGCGCTTGGCTTTTTGAACGAGGACCATTGGGTGGCCAGAGACAATTTCCCATGCGGTGTGGGAGGAGTAG	130	NC_030400.1
NAeflaSw	AACTATCTGGTACATGTGGCTCGGAAGTCGTTTTCTGGAATTTGAGGCCTTGGGGTTCCTAAATGCTGATCACTGGGTCAGTCGTGAAAACTTTCCTGGGGGCGTGGGTGGAGTGGGTGTCAATTACTTT	130	MT577804.1

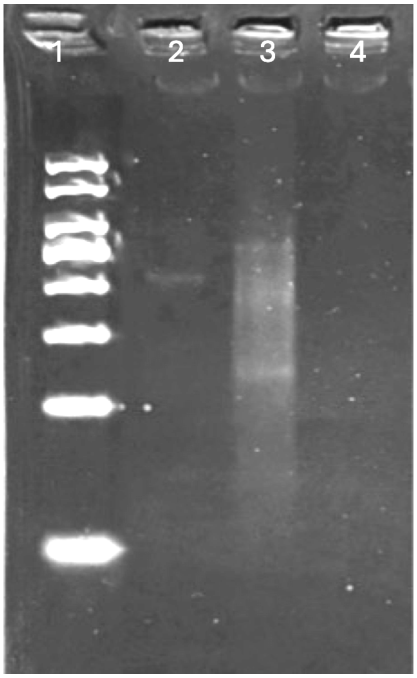

Figure S1: Detection of synthetic double-stranded oligonucleotides by polymerase chain reaction (PCR) using primer XF-F2 and XF-R (215 bp amplicon), visualized on a 2% agarose gel with TBE buffer. Well assignments: (1) 50 bp DNA ladder (Vivantis, Malaysia); (2) Den2Th oligonucleotide – positive control used for technique optimization (Figure 2); (3) Lammi oligonucleotide – one of the negative controls (Figure 3); (4) No template control (NTC). Non-relevant or empty wells were excluded from the image.

Table S3: List of broadly specific orthoflavivirus analyzed in this study.

Primer	Sequence	Gene	bp	Detection technique	Reference
cFD2	GTGTCCCAGCCGGCGGTGTCATCAGC	NS5	262	PCR, fragmentation, microarray	Kuno (1998)
MA	CATGATGGGRAARAGRGARRAG				
XF-F1 (1st round)	AACATGATGGGVAARMGWGARAA	NS5	264	Heminested RT-PCR	Xue et al. (2021)
XF-R	GTRTCCCANCCDGCDGTRTCATCNGC				
XF-F2 (2nd round)	AARGGMAGYMGNGCHATHTGGT	NS5	215		
XF-R	GTRTCCCANCCDGCDGTRTCATCNGC				
PFlav-fAAR	TACAACATGATGGGAAAGAGAGAGAARAA	NS5	267	RT-qPCR, Microarray	Vina-Rodriguez et al. (2017)
PFlav-rKR	GTGTCCCAKCCRGCTGTGTCATC				
FG1	TCAAGGAACTCCACACATGAGATGTACT	NS5	958	Duplex-RT-PCR	Bronzoni et al. (2005); Fulop et al. (1993)
FG2	GTGTCCCATCCTGCTGTGTCATCAGCATACA				
F8276d-F (Flav100F)	AAYTCNACNCANGARATGTAY	NS5	804-830	RT-PCR, Microarray	Grubaugh et al. (2013); Maher-Sturgess et al. (2008)
F9063d-R (Flav200R)	CCNARCCACATRWACCA				
Pan-flavi virus FW	AGNRCYATCTGGTAYATGTGGYTNGG	NS5	1081	RT-PCR	Daidoji et al. (2021)
Pan-flavivirus RV	BHAGCATGTCBTCHGTBGTCATCCA				
MAMD-F	AACATGATGGGRAARAGRGARAA	NS5	264	Heminested RT-PCR	Scaramozzino et al. (2001); Kuno (1998)
FS778-F	AARGGHAGYMCDGCHATHTGGT				
cFD2	GTGTCCCAGCCGGCGGTGTCATCAGC				
FLAVI-NS5fwd-1	GAAATTAATACGACTCACTATAG GGGTACAACATGATGGGGAARAGAGARAARAA	NS5		SHERLOCK	Myhrvold et al. (2018)
FLAVI-NS5rev-1	CGKGTGTCCCAGCCNGCKGTGTCATCWGCA				
Flavi-For	GCMATHTGGTWCATGTGG	NS5	180	RT-PCR	Johnson et al. (2010)
Flavi-Rev	GTRTCCCAKCCDGCNGTRTC				

Primer	Sequence	Gene	bp	Detection technique	Reference
FG1	TCAAGGAACTCCACACATGAGATGTACT	NS5	958	Duplex-RT-PCR	Fulop et al. (1993)
FG2 (Fulop)	TGTATGCTGATGACACAGCAGGATGGGACAC				
YF1	GGTCTCCTCTAACCTCTAG	NS5	675	RT-PCR	Rice et al. (1985); Tanaka (1993)
YF3	GAGTGGATGACCACGGAAGACATGC				
EMF1	TGGATGACSACKGARGAYATG	NS5	500-700	RT-PCR	Pierre et al. (1994)
VD8	GGGTCTCCTCTAACCTCTAG				
Flavi all S2	TACAACATGATGGGMAAACGYGARAA	NS5	264	RT-qPCR	Patel et al. (2013)
Flavi all AS4	GTGTCCCAGCCNGCKGTRTCRTC				
FlaviF1	ATGGCHATGACWGACAC	NS5	352	RT-qPCR	Bonnet et al. (2022)
FlaviF2	CYNTTYCCCATCATGTTNTA				
F5015-F	GTGGTTGGNCTGTATGGNAA	NS3	850	RT-PCR, Microarray	Grubaugh et al. (2013)
F5807-R	CCCATTTCTGAGATGTCAGT				
F1269-F	GAGGCTGGGGAAATGGCTG	E	969	RT-PCR, Microarray	Grubaugh et al. (2013)
F2225-R	CCTCCAACTGATCCAAAGTCCCA				

References

Bonnet, E., van Jaarsveldt, D. and Burt, F.J. 2022. Rapid reverse transcriptase recombinase polymerase amplification assay for flaviviruses using non-infectious in vitro transcribed RNA as positive controls. Journal of Virological Methods 299, 114351.

Bronzoni, R.V.d.M., Baleotti, F.G., Nogueira, R.M.R., Nunes, M. and Figueiredo, L.T.M. 2005. Duplex Reverse Transcription-PCR Followed by Nested PCR Assays for Detection and Identification of Brazilian Alphaviruses and Flaviviruses. J Clin Microbiol 43(2), 696-702.

Daidoji, T., Morales Vargas, R.E., Hagiwara, K., Arai, Y., Watanabe, Y., Nishioka, K., Murakoshi, F., Garan, K., Sadakane, H. and Nakaya, T. 2021. Development of genus-specific universal primers for the detection of flaviviruses. Virology Journal 18(1), 187.

Fulop, L., Barrett, A.D.T., Phillpotts, R., Martin, K., Leslie, D. and Titball, R.W. 1993. Rapid identification of flaviviruses based on conserved NS5 gene sequences. Journal of Virological Methods 44(2), 179-188.

Johnson, N., Wakeley, P.R., Mansfield, K.L., McCracken, F., Haxton, B., Phipps, L.P. and Fooks, A.R. 2010. Assessment of a Novel Real-Time Pan-Flavivirus RT-Polymerase Chain Reaction. Vector-Borne and Zoonotic Diseases 10(7), 665-671.

Kuno, G. 1998. Universal diagnostic RT-PCR protocol for arboviruses. Journal of Virological Methods 72(1), 27-41.

Grubaugh, N.D., McMenamy, S.S., Turell, M.J. and Lee, J.S. 2013. Multi-Gene Detection and Identification of Mosquito-Borne RNA Viruses Using an Oligonucleotide Microarray. PLOS Neglected Tropical Diseases 7(8), e2349.

Maher-Sturgess, S.L., Forrester, N.L., Wayper, P.J., Gould, E.A., Hall, R.A., Barnard, R.T. and Gibbs, M.J. 2008. Universal primers that amplify RNA from all three flavivirus subgroups. Virology Journal 5(1), 16.

Myhrvold, C., Freije, C.A., Gootenberg, J.S., Abudayyeh, O.O., Metsky, H.C., Durbin, A.F., Kellner, M.J., Tan, A.L., Paul, L.M., Parham, L.A., Garcia, K.F., Barnes, K.G., Chak, B., Mondini, A., Nogueira, M.L., Isern, S., Michael, S.F., Lorenzana, I., Yozwiak, N.L., MacInnis, B.L., Bosch, I., Gehrke, L., Zhang, F. and Sabeti, P.C. 2018. Field-deployable viral diagnostics using CRISPR-Cas13. Science 360(6387), 444-448.

Patel, P., Landt, O., Kaiser, M., Faye, O., Koppe, T., Lass, U., Sall, A.A. and Niedrig, M. 2013. Development of one-step quantitative reverse transcription PCR for the rapid detection of flaviviruses. Virology Journal 10(1), 58.

Pierre, V., Drouet, M.T. and Deubel, V. 1994. Identification of mosquito-borne flavivirus sequences using universal primers and reverse transcription/polymerase chain reaction. Research in Virology 145, 93-104.

Rice, C.M., Lenches, E.M., Eddy, S.R., Shin, S.J., Sheets, R.L. and Strauss, J.H. 1985. Nucleotide Sequence of Yellow Fever Virus: Implications for Flavivirus Gene Expression and Evolution. Science 229(4715), 726-733.

Scaramozzino, N., Crance, J.M., Jouan, A., DeBriel, D.A., Stoll, F. and Garin, D. 2001. Comparison of flavivirus universal primer pairs and development of a rapid, highly sensitive heminested reverse transcription-PCR assay for detection of flaviviruses targeted to a conserved region of the NS5 gene sequences. J Clin Microbiol 39(5), 1922-1927.

Tanaka, M. 1993. Rapid identification of flavivirus using the polymerase chain reaction. Journal of Virological Methods 41(3), 311-322.

Vina-Rodriguez, A., Sachse, K., Ziegler, U., Chaintoutis, S.C., Keller, M., Groschup, M.H. and Eiden, M. 2017. A Novel Pan-*Flavivirus* Detection and Identification Assay Based on RT-qPCR and Microarray. BioMed Research International 2017, 4248756.

Xue, Z., Zhao, N., Wang, J., Song, X., Meng, F., Liang, W., Zhou, J., Wang, D., Zhang, Z. and Liu, Q. 2021. Establishment and Application of Heminested RT-PCR Assay for Detection of Mosquito-Borne Flavivirus - Guizhou Province, China, 2018. China CDC weekly 3(1), 4-9.

Table S4: Reference orthoflaviviruses from Table 1 for RPA primer design.

No.	Selected reference pathogenic Orthoflavivirus	No.	Selected reference non-pathogenic Orthoflavivirus
a	Dengue virus type 1 Thailand strain KDH0026A (HG316481.1; 2010)	ba	Lammi virus from Finland (FJ606789.2; 2004)
b	Dengue virus 2 Thailand strain DENV-2/TH/BID-V2617/1996 (FJ906958.1; 1996)	bb	Lammi virus from Finland isolate M0719 (KC692068.1; 2007)
c	Dengue type 3 strain H87 (M93130.1; 1956)	bc	Nakiwogo virus strain Uganda08 (NC_030400.1; 2008)
d	Dengue virus type 4 strain 814669 (AF326573.1; 1981)	bd	Aedes flavivirus from Switzerland strain AEFV/Ticino/2019 (MT577804.1; 2019)
e	Dengue virus type 2 strain TSV01 (AY037116.1; 1993)	be	Royal Farm virus from Afghanistan (NC_039219.1; 1972)
f	Dengue virus type 1 strain Nauru Island, Western Pacific (U88536.1; 1974)	bf	Kamiti River virus isolate SR-82 (NC_005064.1; 1999)
g	Dengue virus type 2 strain 16681 (U87411.1, 1964)	bg	Culex theileri flavivirus from Indonesia strain JKT-8650 (NC_040682.1; 1981)
h	Zika virus Thailand strain SI-BKK01 (KY272987.1; 2016)	bh	Quang Binh virus isolate VN180 (NC_012671.1; 2002)
i	Zika virus from French Polynesia strain Pf13/251013-18 (KY766069.1; 2013)	bi	Palm Creek virus isolate 56 (NC_033694.1; 2010)
j	Zika virus Uganda strain MR 766 (AY632535.2; 1947)	bj	Calbertado virus isolate CALper (KX669689.1; ~2016)
k	Yellow fever virus strain 17D vaccine (X03700.1; 1927)	bk	Chaoyang virus from China isolate HLD115 (NC_017086.1; 2010)
l	Yellow fever virus strain Uganda 2010 (JN620362.1; 2010)	bl	Chaoyang virus from South Korea strain ROK144 (JQ068102.1; 2003)
m	Japanese encephalitis virus strain Vellore P20778 (AF080251.1; 1958)	bm	Donggang virus isolate DG0909 (NC_016997.1; 2009)
n	Japanese encephalitis virus strain JaOArS982 (M18370.1; ~1987)	bn	Nounane virus strain Nounane_B3 (NC_033715.1; 2004)
o	West Nile virus from South Africa strain SA93/01 (EF429198.1; 2001)	bo	Barkedji virus isolate 363/11 (KC496020.1; 2011)
p	West Nile virus from USA strain NY99 (NC009942.1; 1999)	bp	Hepatitis G virus strain HGVCN (U94695.1; ~1996)
q	West Nile virus (M12294.2; ~1979)		
r	Wesselsbron virus from South Africa strain AV259 (JN226796.1; 1997)		
s	Wesselsbron virus from South Africa strain SAH117 (EU707555.1; ~2008)		
t	Murray Valley encephalitis virus strain MVE-1-51 (AF161266.1; ~1999)		
u	Cacipacore virus strain BeAn 3276000 (KF917536.1; 1977)		
v	Duck Tembusu virus from Thailand strain DK/TH/CU-1 (MH460536.1; 2015)		
w	Tembusu virus strain JS804 (JF895923.2; 2010)		

**No.**	**Selected reference pathogenic Orthoflavivirus**	**No.**	**Selected reference non-pathogenic Orthoflavivirus**
x	Usutu virus strain SAAR-1776 (AY453412.1; 1959)		
y	Usutu virus strain Vienna 2001 (NC_006551.1; 2001)		
z	St. Louis encephalitis virus strain Kern217 (DQ525916.1; 1989)		
aa	Bagaza virus strain DakAr B209 (AY632545.2; 2004)		
ab	Ntaya virus isolate IPDIA (JX236040.3; 1966)		
ac	Ilheus virus strain Original (AY632539.4; 1944)		
ad	Kunjin virus strain MRM61C (D00246.1; 1960)		
ae	Langat virus strain TP21 (EU790644.1; 1956)		
af	Tick-borne encephalitis virus (NC_001672.1; 1971)		
ag	Louping ill virus strain 369/T2 (NC_001809.1; 1929)		
ah	Bussuquara virus strain BeAn 4073 (AY632536.4; 1956)		
ai	Kadam virus strain Amp6640 (NC_033724.1; 1967)		
aj	Edge Hill virus strain YMP 48 (DQ859060.1; 2000)		
ak	Sepik virus strain MK7148 (DQ837642.1; 1966)		
al	Kokobera virus strain AusMRM 32 (AY632541.4; 1960)		
am	Jugra virus strain P-9-314 (DQ859066.1; 1969)		
an	Saboya virus strain Dak AR D4600 (DQ859062.1; 1968)		
ao	Potiskum virus strain IBAN 10069 (DQ859067.1; ~1966)		
ap	Bouboui virus strain DAK AR B490 (DQ859057.1; 1967)		
aq	Banzi virus strain SAH 336 (DQ859056.1; 1956)		
ar	Uganda S virus (DQ859065.1; 1971)		
as	Kedougou virus strain DakAar D1470 (AY632540.2; 1972)		

Note: The NCBI accession number and the collection year are indicated in brackets following the virus strain name. A tilde (~) denotes the first online record of that particular sequence.

Table S5: *In-silico* analysis of primer specification.

Primer	Size (base)	GC (%)	Tm min (°C)	Tm mean (°C)	Tm max (°C)	Repeated base
XF-F1	23	39.9	51	55.3	59.4	4
XF-F2	22	50.8	50.5	59.4	68.7	3
XF-R	26	60.3	58.8	64.3	70.9	3
YF1	19	52.6	-	50.3	-	2
FlaviPath-F	30	48.3	55.3 ºC	62.6 ºC	70.4 ºC	3
FlaviPath-R	30	50.6	52.9 ºC	62.3 ºC	73.1 ºC	3

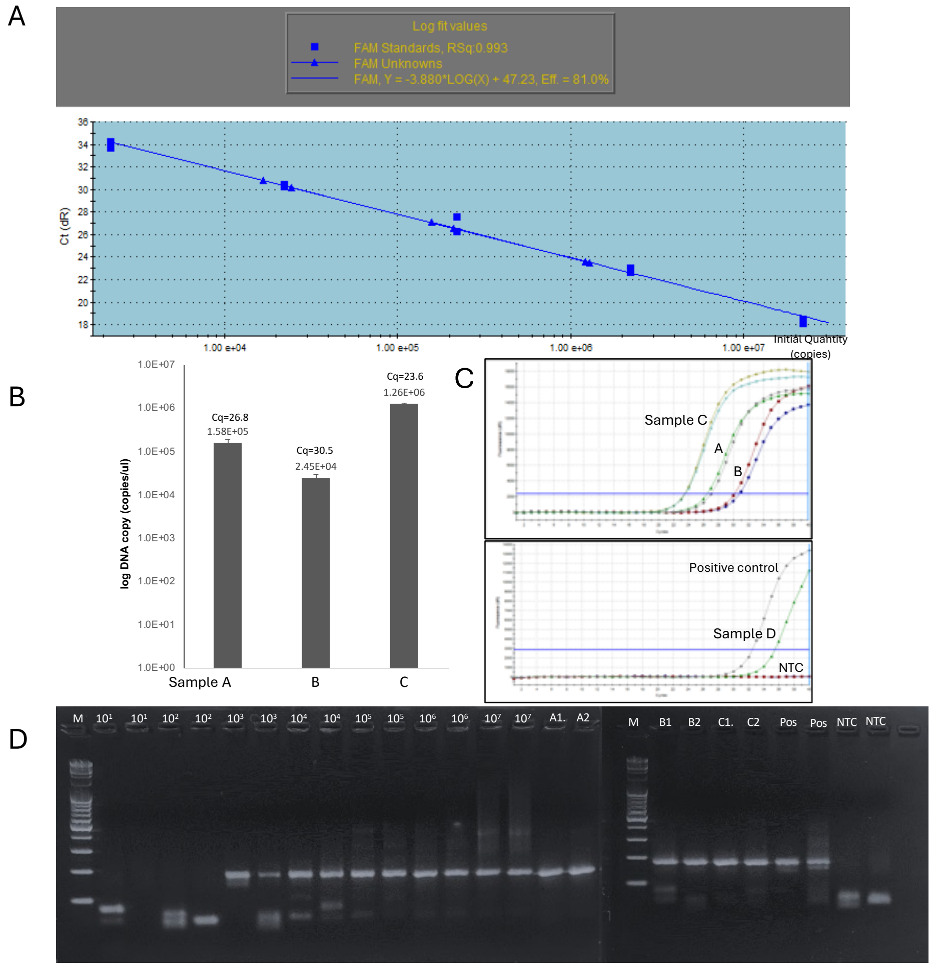

Figure S2: RT-qPCR analysis of dengue virus-infected mosquito samples (sample A, B, C, and D) corresponding to Figure 3 and 4.

A: Calibration curve generated from an RNA standard synthesized via *in situ* transcription from a dengue serotype 2 sample. B: Absolute dengue virus concentrations in mosquito A, B, and C. C: Amplification plots of sample A, B, C, and D. D: Agarose gel electrophoresis of qPCR products (177 bp) amplified using DENV-2 NS5-F and DENV-2 NS5-R primers. Each sample was amplified in technical duplicates and loaded into two wells. The positive control was RNA extracted from a confirmed DENV-2 positive sample. A VC 100 bp plus ladder (Vivantis Technologies, Malaysia) was used as a molecular weight marker. The limit of detection (LOD) was defined as the lowest concentration within the linear calibration range that exhibited the correct melt peak and band position in both the dissociation curve and agarose gel (LOD = 2.2×10^3 copy/µL, Cq = 34.0 cycles). No-template controls (NTC) and the 2.2×10^2 copy/µL standard yielded Cq values around 35.7, which were considered below the LOD threshold. These samples showed no dissociation peaks matching other standards or positive samples (see Fig. S1C) and no visible bands on the agarose gel (see Fig. S2D). Minor signals detected in the qPCR analysis of the NTC and low-copy standard were attributed to primer-dimer formations inherent to the Sybr green detection system. The DNA concentrations measured by the NanoDrop One (Thermo Fisher Scientific) for samples A, B, C, and D, were 67.9, 83.2, 68.1, and 95.3 ng/µL, respectively.

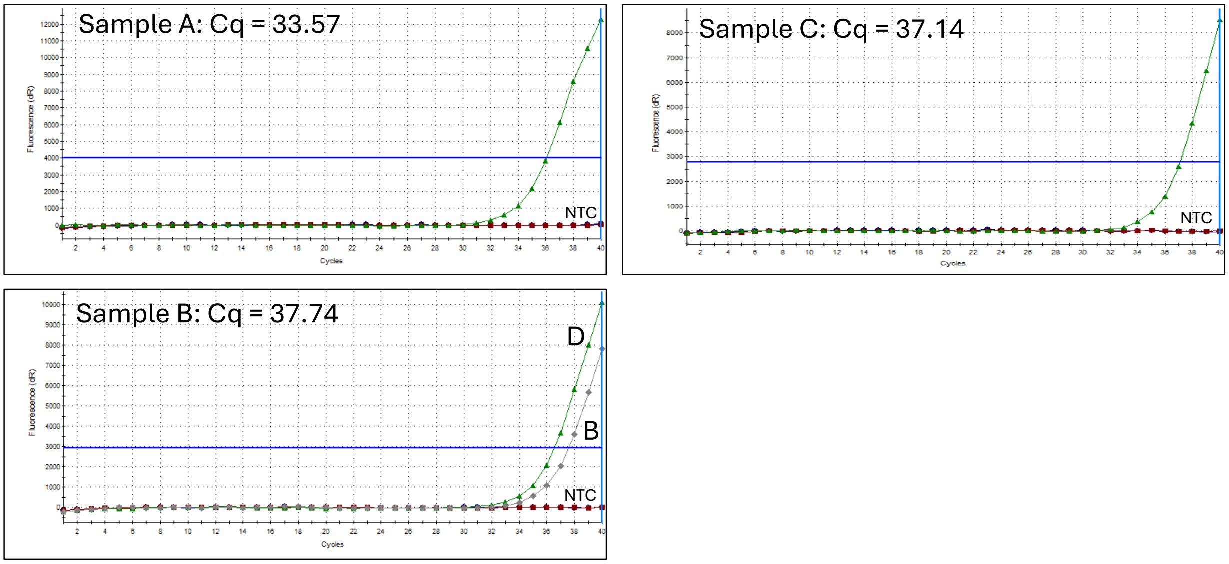

Figure S3: Amplification plots in the RT-qPCR analysis of Zika-infected mosquito samples (A–D) shown in Figure 3. The results were obtained using ZIKF and ZIKR primers. RNA concentrations for samples A, B, and C were 62, 28, and 16 ng/L, respectively (RNA concentration of sample D was unavailable).

Table S6: Normalized intensity raw data used for Figure 2–4

Figuree	Normalized intensity

2

No.	Strand DNA		
	Single (SS)	Double (DS)	NTC
1	0.024	1.072	-0.053
2	0.081	0.202	-0.005
3	0.061	0.221	-

No.	Time (min)					
	5	10	15	20	25	NTC
1	0.105	0.124	0.133	0.207	0.135	0.027
2	0.022	0.036	0.031	0.046	0.039	0.000

No.	Temperature (°C)						
	15	20	25	30	37	42	NTC
1	0.011	0.171	0.183	0.176	0.313	-0.002	0.008
2	-0.003	0.316	0.444	0.235	0.362	0.037	-0.015
3	0.095	0.088	0.088	0.156	0.225	0.063	0.009

3

No.	DNA virus							
	DENV	JEV	ZIKV	WNV	AEFV	NAKV	LAMV	NTC
1	0.693	0.273	0.428	0.182	-0.003	0.027	0.006	-0.005
2	0.256	0.217	0.305	0.379	0.005	-0.005	0.032	0.017

No.	RNA virus			
	DENV	ZIKV	Neg	NTC
1	0.433	1.089	0.011	0.002
2	0.598	0.446	0.001	0.012
3	0.426	0.237		0.008
4	0.228	0.118		

4

No.	Den 2 concentration copies/μL (Mosquito sample)								
	10^0 (A)	10^1 (A)	10^2 (A)	10^3 (A)	10^4 (A)	10^5 (A)	10^4 (B)	10^6 (C)	NTC
1	0.053	0.770	0.025	0.019	0.195	0.141	0.155	0.223	0.007
2	0.216	0.075	0.029	0.016	0.195				-0.005
3	0.069	0.048	0.047	0.052	0.079				0.017
4	0.045	0.259	0.088	0.077	0.036				0.020
5	0.015	0.170	0.062	0.065	0.090				0.016
